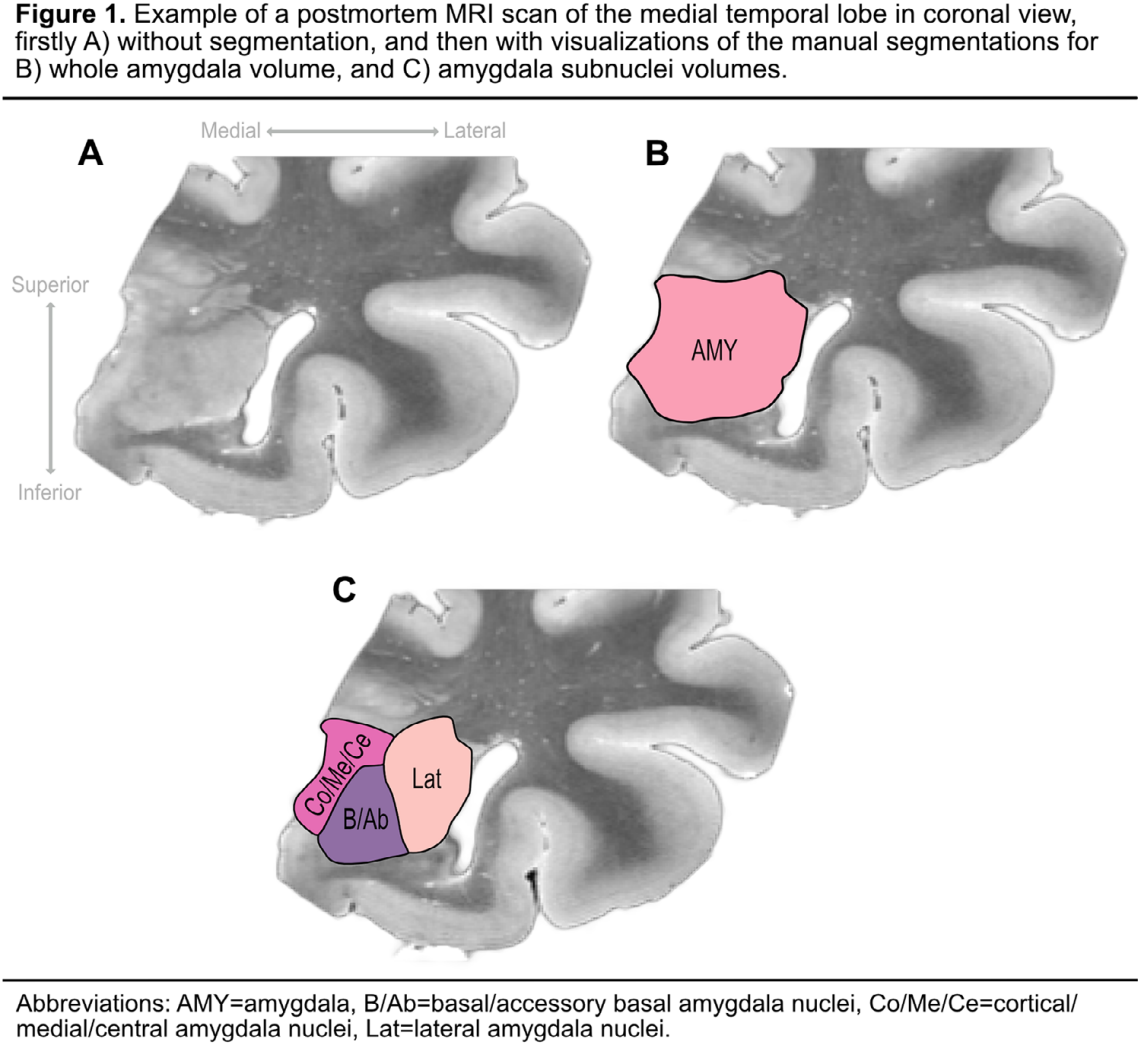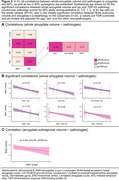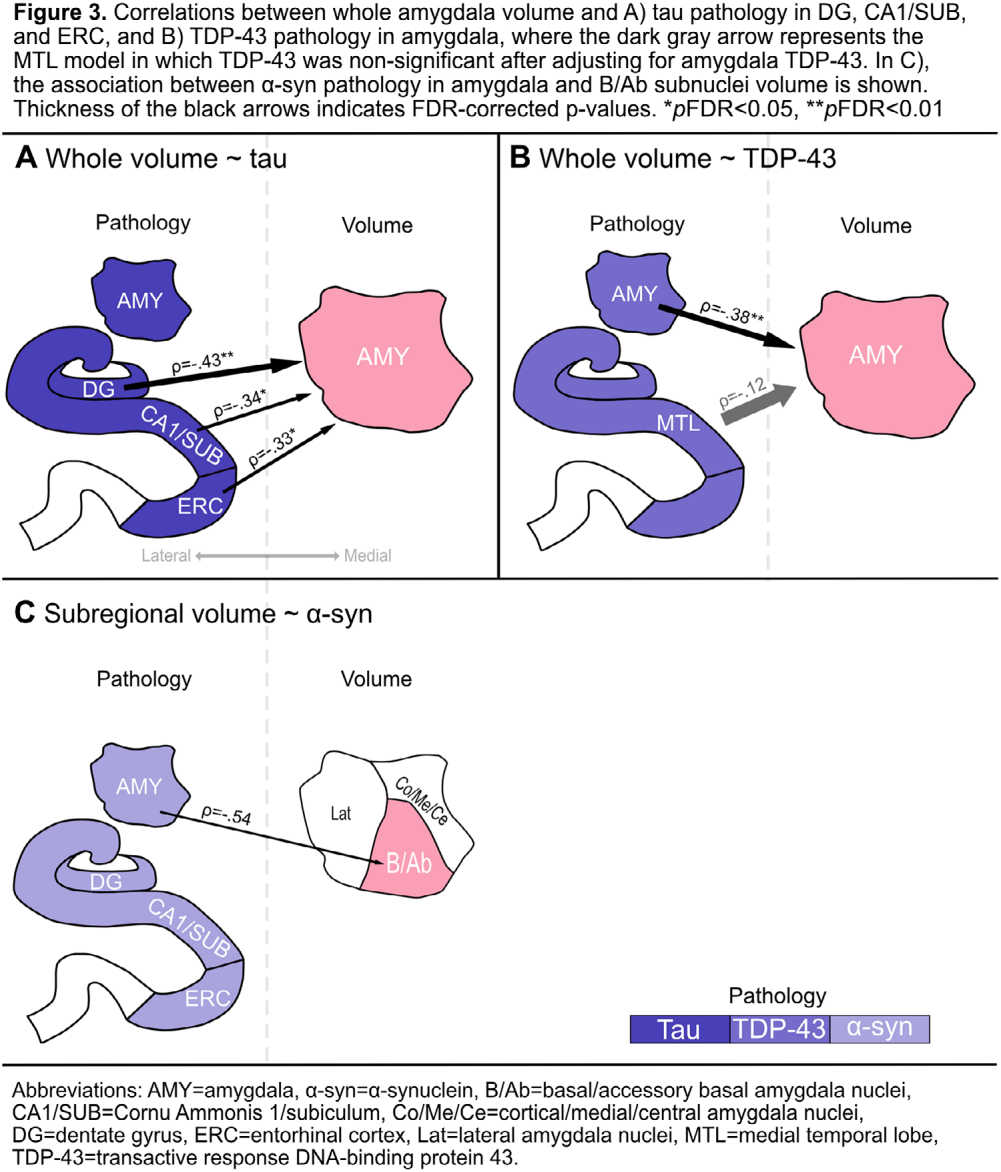# Mapping local and distal effects of different neuropathologies on amygdala volume

**DOI:** 10.1002/alz.087367

**Published:** 2025-01-09

**Authors:** Amanda Annettesdotter, Katja Selezneva, Amanda E Denning, Anika Wuestefeld, Sadhana Ravikumar, Ranjit Ittyerah, Lisa M Levorse, Madigan Bedard, Eunice Chung, John L. Robinson, Theresa Schuck, Eddie B Lee, Sydney A Lim, Winifred Trotman, Alejandra Bahena, David J Irwin, Maria del Mar Arroyo Jimenez, Alicia Vela, Esther Buendia, Maria Mercedes Iniguez de Onzono Martin, Maria del Pilar Marcos Rabal, Monica Munoz, Nicola Spotorno, Oskar Hansson, Ricardo Insausti, Paul A. Yushkevich, Laura E.M. Wisse

**Affiliations:** ^1^ Lund University, Lund Sweden; ^2^ University of Pennsylvania, Philadelphia, PA USA; ^3^ Perelman School of Medicine at the University of Pennsylvania, Philadelphia, PA USA; ^4^ University of Castilla‐La Mancha, Albacete Spain; ^5^ Memory Clinic, Skåne University Hospital, Malmö Sweden; ^6^ Department of Clinical Sciences Lund, Lund University, Lund, Lund Sweden

## Abstract

**Background:**

The amygdala is a hotspot for neuropathologies; however, it is unclear 1) which neuropathologies lead to amygdala neurodegeneration, 2) what specific amygdala subnuclei are affected, and 3) if the neuropathologies related to amygdala volume are local (inside the amygdala), or distal (in other regions). We investigate the relationships between different neuropathologies (tau, amyloid‐β [Aβ], α‐synuclein [α‐syn], and transactive response DNA‐binding protein 43 [TDP‐43]) and amygdala volumes.

**Method:**

We analyzed postmortem data from 73 individuals with and without neurodegenerative diseases (age: 77±11 [45–101] years; 26 [36%] females; 51 [70%] cognitively impaired). Volumes of the whole amygdala and three amygdala subregions (lateral, B/Ab [basal/accessory basal], Co/Me/Ce [cortical/medial/central] nuclei: for n=24 individuals) were manually segmented on 0.2x0.2x0.2 mm^3^ postmortem magnetic resonance images (Figure 1). We used semiquantitative scores (0–3) of Aβ, tau, α‐syn, and TDP‐43 in the amygdala, the dentate gyrus (DG), the Cornu Ammonis 1 and subiculum (CA1/SUB), the entorhinal cortex (ERC), and averaged between the medial temporal lobe (MTL) regions (excluding the amygdala). Partial Spearman correlations were performed to investigate the relationships between neuropathologies and amygdala volumes, adjusting for age, sex, and other pathologies.

**Results:**

Higher ratings of amygdala TDP‐43, MTL TDP‐43, and MTL tau were associated with smaller whole amygdala volume (Figure 2A–B, Figure 3A). However, MTL TDP‐43 was no longer significant when adjusting for amygdala TDP‐43 (Figure 3B). As MTL tau was associated with amygdala volume, we looked into which MTL subregions could be responsible for this association. Here, tau in DG, CA1/SUB, and ERC were all negatively associated with whole amygdala volume (Figure 2B). For exploratory subregional analyses, amygdala α‐syn was initially associated with B/Ab volume (Figure 2C, Figure 3C), but this correlation did not survive FDR‐correction.

**Conclusions:**

These preliminary results indicate that TDP‐43 has a local effect on whole amygdala volume, whereas tau seems to affect amygdala volume distantly through other MTL regions, with the largest effect seen in DG tau, followed by CA1/SUB and ERC. Subregional analyses will be updated once a larger sample is available, as well as further analyses on non‐MTL regions.